# Use of PELC/CpG Adjuvant for Intranasal Immunization with Recombinant Hemagglutinin to Develop H7N9 Mucosal Vaccine

**DOI:** 10.3390/vaccines8020240

**Published:** 2020-05-21

**Authors:** Ting-Hsuan Chen, Chung-Chu Chen, Ming-Hsi Huang, Chung-Hsiung Huang, Jia-Tsrong Jan, Suh-Chin Wu

**Affiliations:** 1 Institute of Biotechnology, National Tsing Hua University, Hsinchu 30013, Taiwan; sadam1114@gmail.com; 2 Department of Internal Medicine, MacKay Memorial Hospital, Hsinchu 30071, Taiwan; 4059@mmh.org.tw; 3 Teaching Center of Natural Science, Minghsin University of Science and Technology, Hsinchu 30401, Taiwan; 4 National Institute of Infectious Diseases and Vaccinology, National Health Research Institutes, Zhunan 35053, Taiwan; huangminghsi@nhri.org.tw; 5 Department of Food Science, National Taiwan Ocean University, Keelung 20224, Taiwan; huangch@mail.ntou.edu.tw; 6 Genomics Research Center, Academia Sinica, Taipei 11529, Taiwan; tsrong33@gate.sinica.edu.tw; 7 Department of Medical Science, National Tsing Hua University, Hsinchu 30013, Taiwan

**Keywords:** H7N9, intranasal, adjuvant, mucosal vaccine

## Abstract

Human infections with H7N9 avian influenza A virus can result in severe diseases with high mortality. Developing an effective vaccine is urgently needed to prevent its pandemic potential. Vaccine delivery routes via mucosal surfaces are known to elicit mucosal immune responses such as secretory IgA antibodies in mucosal fluids, thus providing first-line protection at infection sites. PEG-b-PLACL (PELC) is a squalene-based oil-in-water emulsion adjuvant system that can enhance antigen penetration and uptake in nasal mucosal layers with enhanced mucin interactions. In this study, intranasal immunizations with recombinant H7 (rH7) proteins with a PELC/CpG adjuvant, as compared to the use of poly (I:C) or bacterial flagellin adjuvant, elicited higher titers of H7-specific IgG, IgA, hemagglutination inhibition, and neutralizing antibodies in sera, and increased numbers of H7-specific IgG- and IgA-antibody secreting cells in the spleen. Both PELC/CpG and poly (I:C) adjuvants at a dose as low as 5 μg HA provided an 80% survival rate against live virus challenges, but a lower degree of PELC/CpG-induced Th17 responses was observed. Therefore, the mucosal delivery of rH7 proteins formulated in a PELC/CpG adjuvant can be used for H7N9 mucosal vaccine development.

## 1. Introduction

Influenza A viruses pose a constant threat to human public health. In 2013, a novel H7N9 influenza virus emerged and caused severe diseases, such as pneumonia and/or acute respiratory distress syndrome [1,2], and deaths in humans [3,4]. These viruses further evolved in 2016–2017 into two lineages: the Yangtze River Delta and Pearl River Delta [5,6,7,8]. Some H7N9 isolates of the Yangtze River Delta lineage were found to contain polybasic amino acids at the hemagglutinin (HA) cleavage sites, which is the signature of highly pathogenic avian influenza viruses [9,10]. Therefore, there is an urgent need to develop H7N9 vaccines to prevent further spread of the virus and to prevent a potential pandemic.

Mucosal vaccinations via mucosal routes such as nasal, sublingual, oral, rectal, or vaginal can elicit mucosal immune responses such as secretory IgA antibodies, thus providing first-line protection at infection sites [11,12]. Nasal delivery is one of the common routes for mucosal immunization, where the antigens associate with nasopharynx-associated lymphoid tissues containing M cells, antigen presenting cells, T cells, and B cells to trigger mucosal immune responses such as IgA-secreting B or plasma cells [13]. To elicit an effective mucosal immunity, nasal immunization requires the use of an adjuvant to break tolerance to the antigen [11,12,13]. Nasal delivery vaccination has been studied extensively using nanoparticles such as polysaccharides (chitosan), polymers (association chitosan polymers, starch polymers), poly (lactic-co-glycolic acid (PLGA), lipids (liposomes, virosomes), immunostimulating complex (ISCOM), and proteins (lipopeptides) [14]. In particular, chitosan with or without PLGA polymers increased permeation and enhanced antigen delivery in mucosa by nasal delivery [15,16]. We recently found that PEG-b-PLACL (PELC), a squalene-based oil-in-water emulsion adjuvant system, can enhance antigen penetration and uptake in nasal mucosal layers with enhanced mucin interactions [17]. PELC is a squalene-in-water emulsion, stabilized by a combination of Span^®^ 85 (sorbitan trioleate) and poly (ethylene glycol)-block-poly (lactide-co-ε-caprolactone), which has a composition similar to MF59, except for the replacement of Tween 80 with a biodegradable PEG-b-PLACL polymer [18].

In this study, we investigated intranasal rH7 immunizations in mice using a combination of PELC and K3 CpG oligodeoxynucleotides (ODN) as a mucosal adjuvant. We previously reported that inactivated H5N1 virions formulated with PELC/CpG elicited more potent immune responses than PELC alone [18]. In parallel, we also conducted intranasal rH7 immunizations in mice with two Toll-like receptor (TLR)-targeting adjuvants: TLR-3 agonist poly (I:C) [19] or TLR5 agonist bacterial flagellin (FliC). Three-dose intranasal immunizations were conducted in BALB/c mice. The titers of IgG, IgA, hemagglutination inhibition (HI), and microneutralization (MN) antibodies were measured in sera and bronchoalveolar lavage fluids (BALFs), as well as the numbers of IgG and IgA antibody-secreting cells (ASCs) and germinal center (GC) B cells in the spleen. Th1, Th2, and Th17 cellular responses were also determined in the spleen and cervical lymph nodes (CLNs). The protective levels for these immunized mice were assessed following lethal virus challenges. Our present findings may provide useful information for H7N9 mucosal vaccine development.

## 2. Results

### 2.1. Intranasal rH7 Immunizations with PELC/CpG, Poly (I:C) or FliC Adjuvants to Elicit H7-Specfic IgG, IgA, and Neutralizing Antibodies in Sera and BALFs

**Figure 1:**
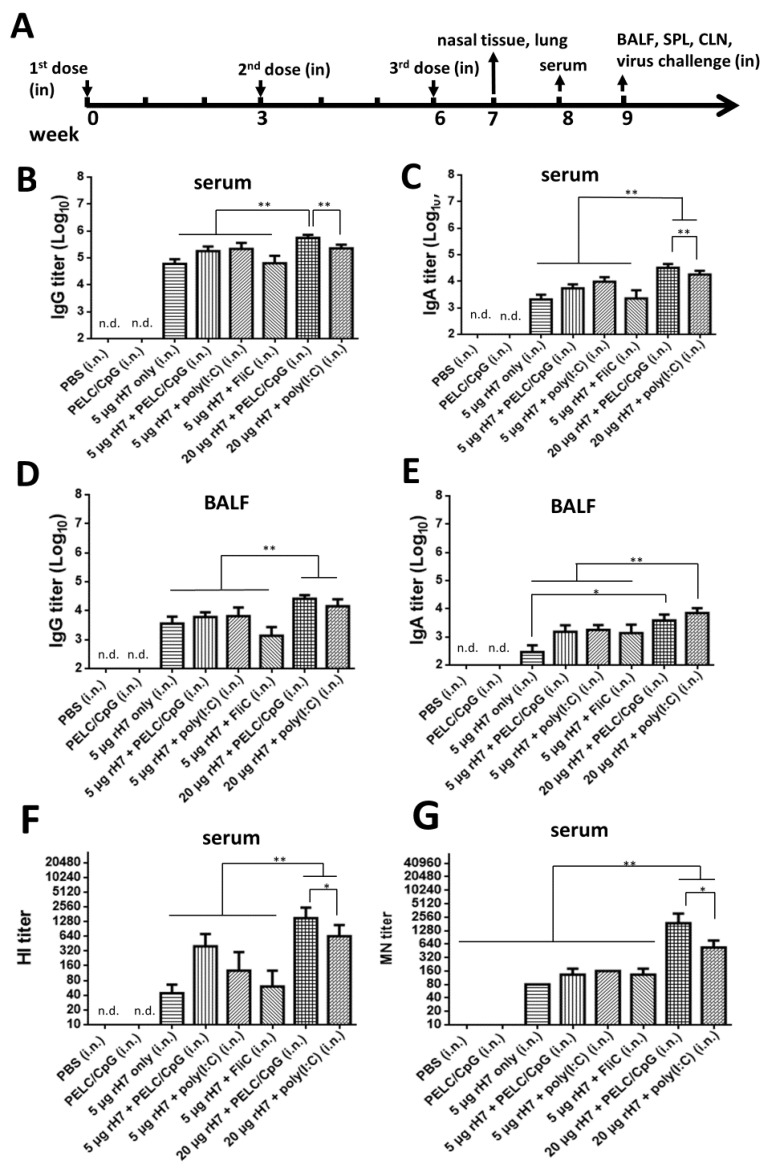
Antibody responses in sera and BALFs elicited by intranasal immunizations with rH7 proteins formulated in PELC nanoemulsion plus CpG. (**A**) Groups of mice were intranasally immunized with three doses of 5 or 20 µg rH7 protein formulated with PELC/CpG, poly (I:C), or FliC over a 3-week interval. Serum and BALF samples were collected and analyzed using ELISA to measure (**B**,**D**) H7-specific IgG and (**C**,**E**) IgA antibody titers. Hemagglutination (HA) inhibition and neutralization antibody titers in sera were respectively determined using (**F**) hemagglutination inhibition (HI) and (**G**) microneutralization (MN) assays. n.d. (not detected). Statistical analysis consisted of one-way ANOVA. *, *p* < 0.05; **, *p* < 0.01.

To develop rH7-based mucosal vaccines, groups of BALB/c mice were intranasally immunized with three doses of 5 or 20 μg rH7 proteins over a 3-week interval with or without the use of (i) PELC/CpG, (ii) poly (I:C), or (iii) FliC adjuvant (Figure 1A). Analyses of sera and BALFs showed that all the rH7-immunized groups elicited significant titers of H7-specific IgG and IgA antibodies compared to the PBS and PELC/CpG adjuvant only control (Figure 1B–E). At the 5 μg dose for rH7 immunizations, the use of the PELC/CpG and poly (I:C) adjuvants resulted in 0.4–0.5 log increased IgG and 0.4–0.6 log increased IgA titers in sera than in the FliC adjuvant (Figure 1B,C). At the 20 μg dose for rH7 immunizations, the use of the PELC/CpG adjuvant elicited even higher IgG (0.4 log increase) and IgA (0.3 log increase) titers in sera, but not BALF, than the use of poly (I:C) (Figure 1B–E). Virus-neutralizing antibodies determined by HI and MN titers in antisera indicated that intranasal immunizations with 5 μg and 20 μg rH7 and the use of the PELC/CpG adjuvant elicited the highest HI and MN titers compared to poly(I:C) and FliC adjuvants (Figure 1F,G). 

### 2.2. B Cell Subsets in the Spleen

**Figure 2:**
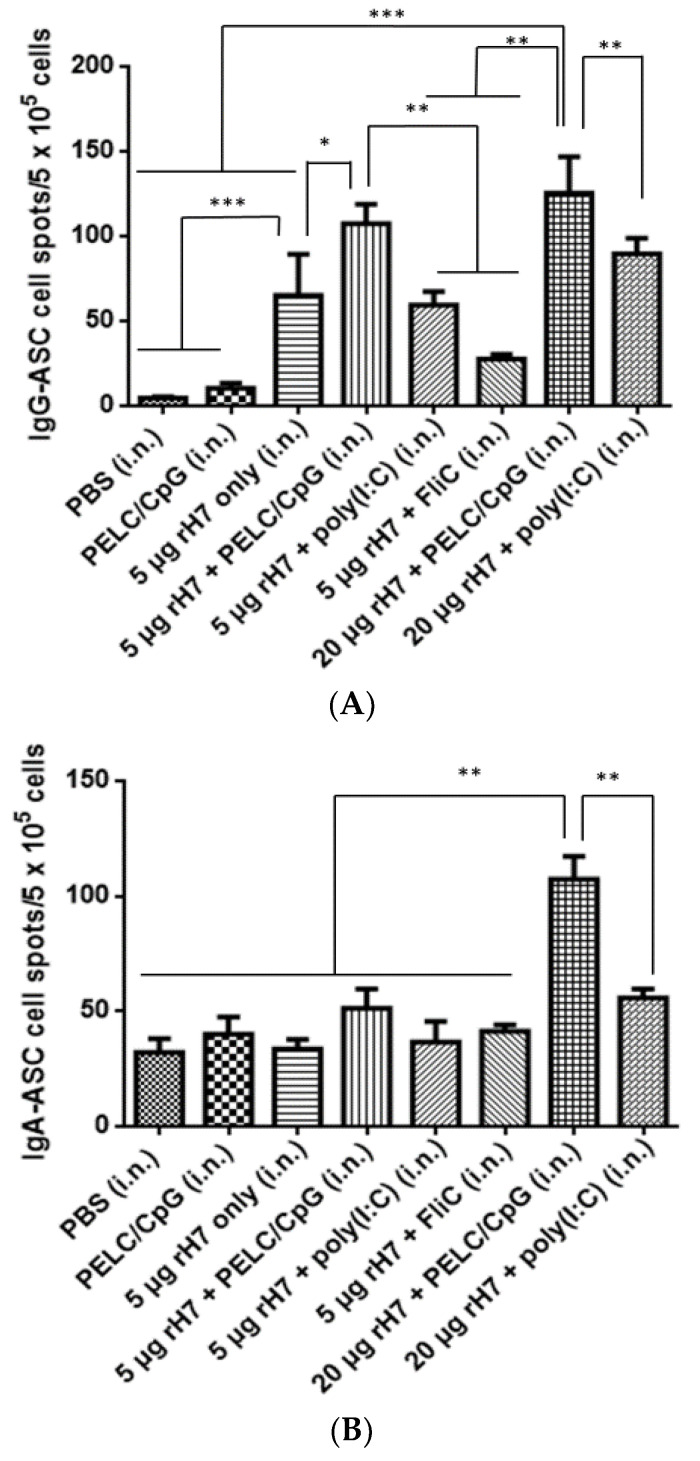

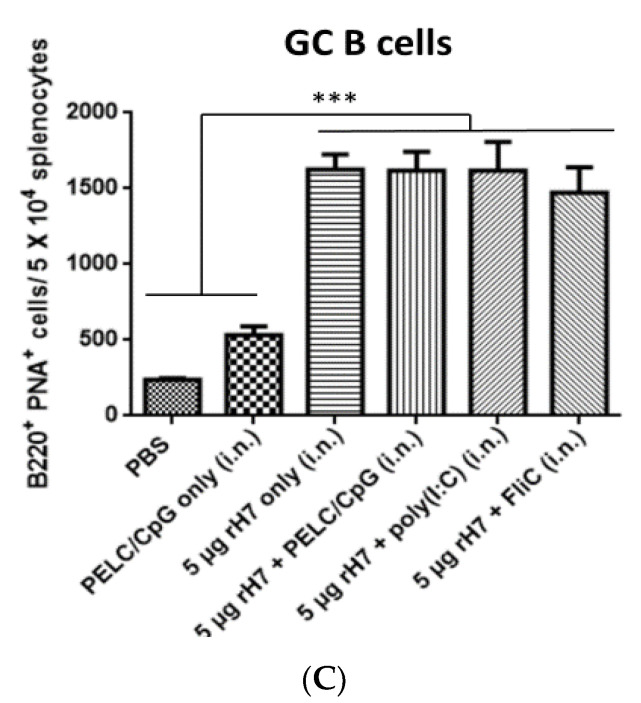
H7-specific IgG-, IgA-ASCs in spleen. Splenocytes isolated from immunized mice were stimulated with rH7 proteins and analyzed using ELISPOT assays to determine H7-specific (**A**) IgG-ASCs and (**B**) IgA-ASCs. (**C**) GC-B cells were determined using flow cytometry for the detection of B220^+^PNA^+^ cells. Statistical analysis consisted of one-way ANOVA. *, *p* < 0.05; **, *p* < 0.01; ***, *p* < 0.001.

Splenocytes were collected at 3 weeks following the third immunization of the mice, stimulated with rH7 proteins, and analyzed with the enzyme-linked immunospot (ELISPOT) assay for IgG- and IgA-ASCs. At the 5 μg dose for rH7 immunizations, the use of the PELC/CpG adjuvant significantly increased the numbers of IgG-ASCs but maintained similar numbers of IgA-ASCs in the spleen, followed by the use of poly (I:C) and FliC adjuvants (Figure 2A,B). At the 20 μg dose rH7 immunizations, the PELC/CpG adjuvant induced higher numbers of IgG-ASCs and IgA-ASCs in the spleen than poly (I:C) (Figure 2A,B). GC B cells from rH7-stimulated splenocytes were also determined using flow cytometry analysis, which showed no differences with or without the use of the PELC/CpG, poly (I:C), or FliC mucosal adjuvant (Figure 2C). Therefore, intranasal rH7 immunizations with the use of the PELC/CpG, compared to poly (I:C) and CpG adjuvants, elicited higher H7-specific IgG- and IgA-ASCs but not GC B cells in the spleen.

### 2.3. T Cell Subsets in the Spleen

**Figure 3:**
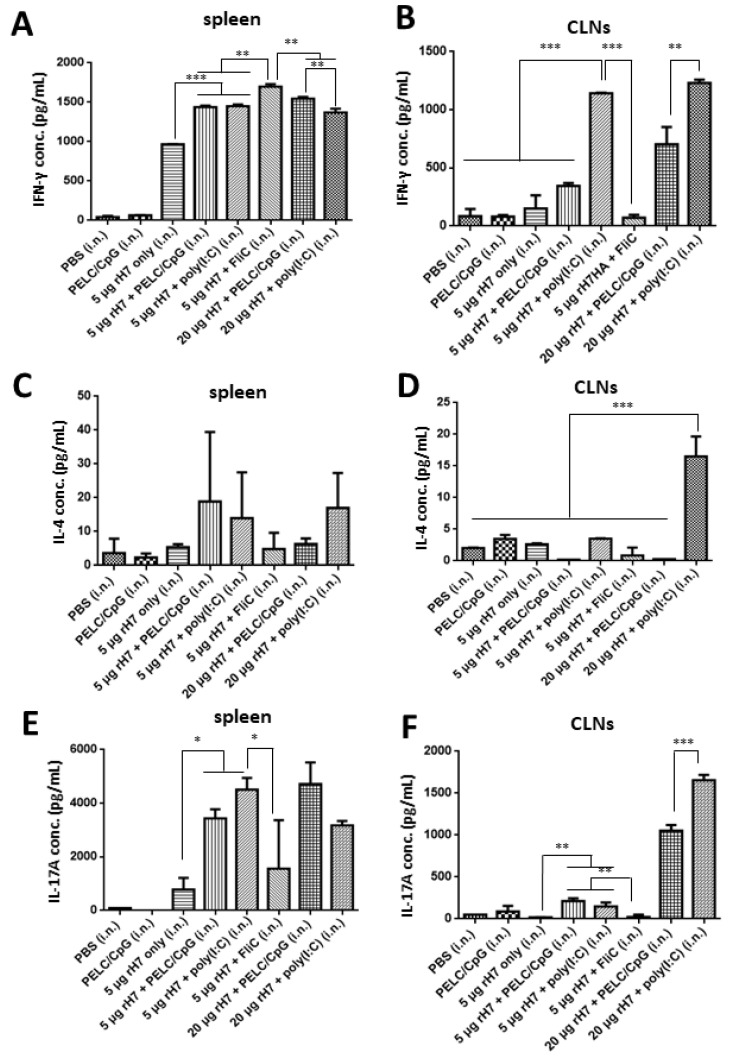
T cell responses in splenocytes and CLNs induced by intranasal immunizations with rH7 proteins. Splenocytes and CLNs collected from immunized mice were cultured and stimulated with rH7 proteins. Culture supernatants were analyzed using ELISA to measure (**A**,**B**) IFN-γ, (**C**,**D**) IL-4, and (**E**,**F**) IL-17A cytokine levels to determine H7-specific Th1, Th2, and Th17 cell activation, respectively. Statistical analysis consisted of one-way ANOVA. *, *p* < 0.05; **, *p* < 0.01; ***, *p* < 0.001.

To determine T cell responses elicited by intranasal rH7 immunizations, with or without the PELC/CpG, poly (I:C) or FliC adjuvant, splenocytes and CLNs were collected 3 weeks after the third dose immunizations, stimulated with rH7 proteins, and analyzed using the enzyme-linked immunosorbent assay (ELISA) to determine IFN-γ, IL-4, and IL-17A production by T cells. Our data indicate significant increases in IFN-γ levels in splenocytes from all mice immunized with 5 μg or 20 μg rH7 with the use of the PELC/CpG, poly (I:C), or FliC adjuvant compared to the rH7 only control (Figure 3A). In CLNs, the use of the poly (I:C) adjuvant resulted in higher IFN-γ levels compared to the use of the PELC/CpG and FliC adjuvants (Figure 3B). For IL-4 secreting T cells, the use of the PELC/CpG and poly (I:C) adjuvant induced more Th2 cells in splenocytes (Figure 3C); only the use of the poly (I:C) adjuvant induced more Th2 cells in CLNs (Figure 3D). For IL-17A secreting T cells, 5 μg dose rH7 immunizations, with or without the use of PELC/CpG, poly (I:C), and FliC adjuvants, resulted in significantly higher titers of IL-17A production from the stimulated splenocytes than those in the PBS- and PELC/CpG-immunized control groups (Figure 3E). In CLNs, the use of the poly (I:C) adjuvant for 20 μg dose rH7 immunizations resulted in higher IL-17A levels than that by the use of the PELC/CpG adjuvant (Figure 3F). 

### 2.4. IHC Staining for IL-17A Production in Lung Tissue Cells 

**Figure 4:**
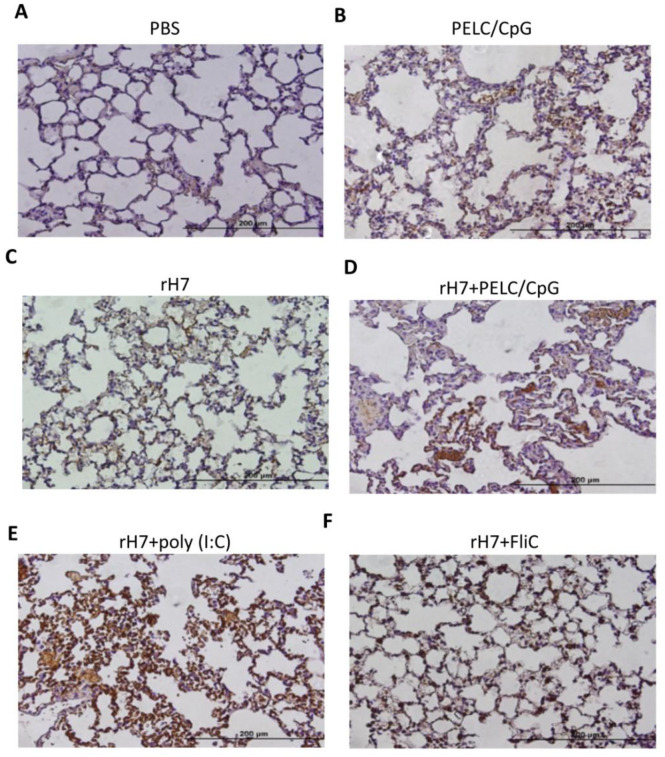
IL-17 expression in lung tissues. One week after the final immunization, lung sections from mice (N = 3 per group) intranasally immunized with (**A**) PBS alone, (**B**) PELC/CpG alone, (**C**) rH7 alone, (**D**) rH7 + PELC/CpG, (**E**) rH7 + poly (I:C), or (**F**) rH7 + FliC were collected and stained with anti-IL-17A antibodies for the observation of IL-17 secretion under an Olympus DP70 microscope (×400 magnification).

To examine the levels of IL-17A in lung tissues intranasally immunized with the use of the PELC/CpG, poly (I:C), or FliC adjuvant, we conducted IHC staining to examine IL-17A production in the lung tissues of immunized mice one week after the third dose of intranasal immunizations. The results showed that the use of the poly (I:C) adjuvant, administered intranasally, induced higher IL-17A levels in lung tissue cells than that by the use of the PELC/CpG adjuvant when administered intranasally (Figure 4). The use of FliC adjuvant, administered intranasally, showed a further lower IL-17A level in lung tissue cells (Figure 4). Therefore, comparatively, the PELC/CpG adjuvant showed lower IL-17A levels in lung tissue cells than did the poly (I:C) adjuvant, but the level of IL-17A was still higher than that by the use of the FliC adjuvant when administered intranasally.

### 2.5. H&E Staining for Nasal Cavity Tissues

**Figure 5:**
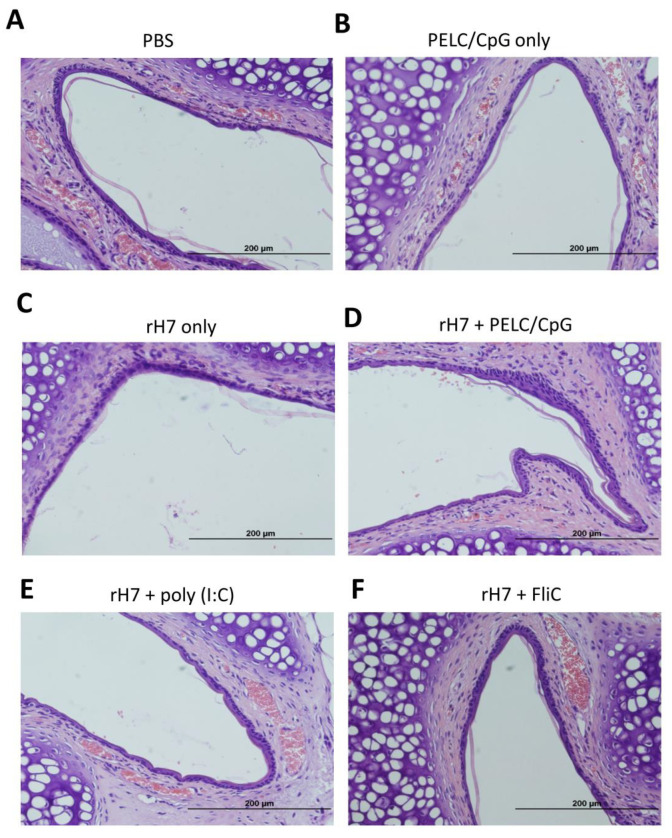
Representative photographs of nasal tissue sections (×400 magnification). Groups of mice (N = 3 per group) were intranasally immunized with (**A**) PBS alone, (**B**) PELC/CpG alone, (**C**) rH7 alone, (**D**) rH7 + PELC/CpG, (**E**) rH7 + poly (I:C), or (**F**) rH7 + FliC. One week after the final vaccination, the mice were sacrificed and the nasal tissue sections collected from mice were stained (H&E) for histopathological examination.

To investigate whether the use of the PELC/CpG, poly (I:C), or FliC adjuvant via the intranasal route may cause acute inflammation at the administration site, we examined the nasal cavity tissues of immunized mice using H&E staining. One week after the third dose of intranasal immunizations, mice were sacrificed and their nasal cavity sections were collected and stained (H&E) for histopathological examination. Our results revealed no inflammation (in the absence of cellular infiltration) in the nasal tissues following the three-dose intranasal rH7 immunizations with the use of the PELC/CpG, poly (I:C), and FliC adjuvants (Figure 5). 

### 2.6. Protective Immunity for rH7 Intranasal Immunization Using PELC/CpG or Poly (I:C) Mucosal Adjuvant

**Figure 6:**
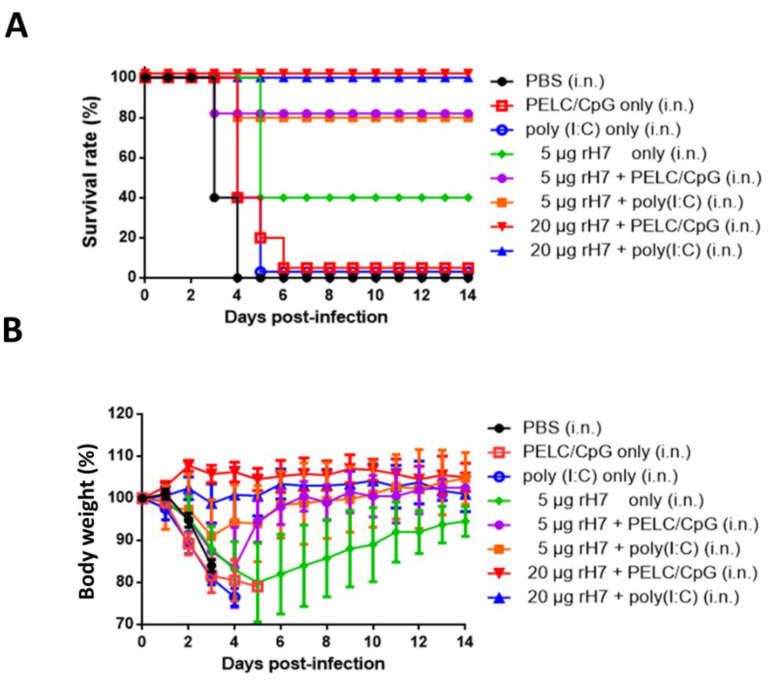
Protective immunity against the H7N9 virus induced by intranasal immunization with rH7 proteins. Mice were challenged with 10 LD_50_ of the H7N9 virus 3 weeks after the final intranasal immunizations. (**A**) Survival and (**B**) body weight loss rates were monitored for 14 days. Mice whose body weights fell below 75% of their initial weights were sacrificed.

To determine the protective immune responses following intranasal immunizations with rH7, with or without the use of the PELC/CpG or poly (I:C) adjuvant, immunized mice were challenged with 10 lethal dose 50% (LD_50_) of A/Taiwan/01/2013(H7N9) viruses 3 weeks after their third dose of immunizations. The results indicate that 20 μg rH7 plus either the PELC/CpG or poly (I:C) adjuvant provided a 100% survival rate of the immunized mice following live virus challenges (Figure 6A). Survival rates decreased to 80% in mice immunized with 5 μg rH7 + PELC/CpG or poly (I:C) adjuvant, to 40% in mice treated with 5 μg rH7 without adjuvant, and to 0% for the PBS-, PELC/CpG only-, or poly (I:C) only-immunized groups (Figure 6A). Few significant differences in body weight recovery were observed in the groups of mice immunized with 5 or 20 μg rH7 plus PELC/CpG or poly (I:C) adjuvant, but a much slower recovery rate was noted for mice in the 5 μg rH7 without adjuvant group (Figure 6B). Zero weight recovery was observed in the PBS-, PELC/CpG adjuvant only-, or poly (I:C) adjuvant only-immunized mice (>25% average body weight loss) (Figure 6B).

## 3. Discussion

An effective mucosal vaccine can elicit not only the systemic immune responses, like serum neutralizing antibodies, but also the mucosal immune responses, such as secretory IgA antibodies in mucosal fluids, to provide first-line protection at the infection sites. In this study, we investigated three mucosal adjuvant systems for intranasal rH7 immunizations, including (i) PELC/CpG, (ii) poly (I:C), and (iii) the natural TLR5 ligand bacterial flagellin FliC adjuvants. Our findings indicate that the use of the PELC/CpG adjuvant, as compared to the use of the poly (I:C) or FliC adjuvant, elicited higher IgG and IgA and neutralizing antibody titers in sera, increased numbers of IgG-, and IgA-ASCs in the spleen. However, the use of the PELC/CpG adjuvant for intranasal rH7 immunization elicited a less potent Th17 cellular response in both the spleen and CLNs compared to the use of the poly (I:C) adjuvant. Both the PELC/CpG and poly (I:C) adjuvants with a 5 μg rH7 dose provided a more than 80% survival rate for the immunized mice following live H7N9 virus challenges.

We have previously demonstrated that intramuscular immunizations with HA proteins formulated with PELC/CpG K3 CpG oligodeoxynucleotides (ODN) can elicit more potent neutralizing antibodies and protective immunity against H5N1 and H7N9 viruses [20,21]. Intranasal immunizations with the PELC oil-in-water emulsion, in addition to LD-indolicidin, have previously been shown to enhance anti-HA serological immunity against influenza viruses [17]. In this study, we investigated the use of PELC/CpG for rH7 intranasal immunizations and compared the stimulatory effects with a poly (I:C) adjuvant. Three-dose immunizations with a three-week interval were conducted to elicit systemic and mucosal IgG and neutralizing antibody titers, since mucosal delivery generally requires a higher antigen content and multiple booster doses to elicit effective immune responses. As TLR3 was reported to be the predominant type in airway epithelial cells [22], intranasal immunizations with the use of poly (I:C) or rintatolimod TLR3 agonists have extensively been reported to elicit potent mucosal IgA antibodies and provide protection against influenza virus challenges in mice, monkeys, and humans [23,24,25,26]. Here, we found that the PELC/CpG adjuvant elicited significantly higher titers of IgG, IgA, HI, and MN than the poly (I:C) adjuvant in sera for intranasal rH7 immunizations (Figure 1). The results also correlate with the increased numbers of IgG- and IgA-ASCs, but not the GC B cells, detected by measuring splenocytes from immunized mice stimulated with rH7 antigens (Figure 2). It is possible that the enhanced IgA secretion observed in BALFs by the use of the PELC/CpG adjuvant may not be due to the promotion of GC formation in secondary lymphoid organs.

We also measured T cell responses in the spleen and CLNs, and the results indicated that using PELC/CpG, compared to poly (I:C), for rH7 intranasal immunizations, induced less potent Th1, Th2, and Th17 cellular responses, particularly in CLNs (Figure 3). We also detected significantly higher IL-17 levels in lung tissue cells with the use of the PELC/CpG or poly (I:C) adjuvant, but the PELC/CpG adjuvant showed a lower level than did the poly (I:C) adjuvant (Figure 4). As Th17 cells may mediate B cell development in the germinal center to enhance the production of secretory IgA in mucosal fluids [27,28,29], the lower degree of PELC/CpG-induced Th17 responses, compared to the use of the poly (I:C) adjuvant, did not reduce the production of secretory IgA in sera (Figure 1C) and BALFs (Figure 1E), as well as the number of IgA-ASCs in the spleen (Figure 2B) in our present studies. Further investigations are required to understand how the PELC/CpG adjuvant mediates the Th17 cellular response to promote secretory IgA production.

Intranasal immunization with rH7 plus the PELC/CpG adjuvant at a dose as low as 5 μg HA provided an 80% survival rate, similar to that offered by the use of the poly (I:C) adjuvant (Figure 6). These results differ from those of other studies on intranasal immunization against H1N1 and H5N1 viruses, including the use of chitosan nanoparticles at 15 µg of HA per dose [30,31] and mucosal co-delivery with a mast cell activator protein at 9 µg of HA per dose [32]. Our results also showed that intranasal immunization with 5 μg rH7 without adjuvant was able to provide a 40% survival rate with the full recovery of weight loss (~95%) (Figure 6), which may be due to the contamination of the rH7 proteins with residual baculoviruses or insect cell-derived proteins to trigger unknown innate pathways in nasal-associated lymphoid tissues (NALTs), as previously reported [33,34]. A previous study reported that the intranasal administration of a chitosan adjuvant was sufficient to elicit the innate immune memory, also as known as “trained immunity,” to provide a complete protection in BALB/c mice following a lethal H7N9 virus challenge [35]. However, our results clearly demonstrated that the administration of the PELC/CpG adjuvant only via intranasal immunizations did not provide any level of protection against challenges (Figure 6). The PELC/CpG adjuvant used for intranasal rH7 immunizations can thus be applied for developing H7N9 mucosal vaccines.

## 4. Conclusions

Intranasal rH7 immunizations with the use of PELC/CpG adjuvant, as compared to poly (I:C) or FliC adjuvant, induced higher IgG and IgA and neutralizing antibody titers in sera, increased numbers of IgG-, and IgA-ASCs in the spleen. Both PELC/CpG and poly (I:C) adjuvants at a dose as low as 5 μg HA provided an 80% survival rate against live virus challenges but a lower degree of PELC/CpG-induced Th17 responses was observed. Therefore, the mucosal delivery of rH7 proteins formulated in a PELC/CpG adjuvant may be used to develop H7N9 mucosal vaccines.

## 5. Materials and Methods

### 5.1. rH7 Protein Expression and Purification

The HA cDNA sequence of the A/Shanghai/2/2013 (H7N9) virus strain was used to construct a plasmid for soluble rH7 proteins. An insect cell codon-optimized H7HA coding sequence was obtained from Genomics BioSci & Tech. Ltd. (New Taipei, Taiwan). The C terminal cytoplasmic and transmembrane domains of full-length HA were deleted and replaced with a GCN4-pII leucine zipper (MKQIEDKIEEILSKIYHIENEIARIKKLIGEV) for trimerization, followed by a thrombin cleavage site, ending with a His-tag for purification as previously described [20]. The Bac-to-Bac System (Invitrogen, Carlsbad, CA, USA) was used to obtain recombinant baculoviruses according to the manufacturer’s instructions. For large-scale production, Sf9 cells were incubated in 600 mL Sf-900 SFM II serum-free medium (Invitrogen) at a concentration of 2 × 10^6^ cells/mL, and then infected with a specific recombinant baculovirus at a multiplicity of infection (MOI) of 3. Culture supernatants were collected 72 h post infection, and then rH7 proteins were purified with nickel-chelated affinity chromatography (Tosoh, Minato-ku, Tokyo, Japan), dialyzed with PBS, and stored at −20 °C.

### 5.2. Mouse Immunization 

For the vaccine preparations, 5 or 20 μg of rH7 proteins formulated with PELC/CpG (10% PELC + 10 μg CpG) [21], 2 μg poly (I:C) (InvivoGen, San Diego, CA, USA) 10 μg R848 (InvivoGen), or 1 μg recombinant flagellin (FliC) [36] were prepared in PBS (30 µL total volume per mouse). Female BALB/c mice (6–8 weeks old, five mice per group) were anesthetized with 30 mg/kg Zoletil 50 (Virbac, Westlake, TX, USA) via intraperitoneal injection prior to each immunization, after which PELC/CpG alone; PBS; 5 μg of rH7 proteins (no adjuvant); 5 μg of rH7 proteins formulated with R848 or FliC; or 5 or 20 μg of rH7 proteins plus PELC/CpG or poly (I:C) were dropped into the nostrils (15 µL vaccine for each nostril) three times over a 3-week interval. Serum samples were collected 2 weeks after the third immunization; splenocytes, CLNs, and BALFs were collected 1 week later. All procedures involving animals were performed in accordance with the guidelines established by the Laboratory Animal Center of National Tsing Hua University (NTHU, Hsinchu, Taiwan). Animal use protocols were reviewed and approved by the NTHU Institutional Animal Care and Use Committee (approval no. 10246). 

### 5.3. H7-Specific IgG and IgA Antibody Titers 

Plates (96-well) were coated with 100 µL of purified rH7 protein (2 µg/mL), and incubated overnight at 4 °C. The plates were washed three times with 300 µL PBST (PBS with 0.05% Tween-20), and then blocked with 200 µL PBS buffer plus 1% BSA for 2 h at room temperature (RT), followed by three additional washes with 300 µL PBST. Two-fold serially diluted serum or BALF samples (from 2^0^–2^7^ × 10^4^ and 2^0^–2^7^ × 10^2^, respectively) were added to each well, followed by a 1 h incubation at RT. After three washes with 300 µL PBST, the plates were incubated with 100 µL horseradish peroxidase (HRP) conjugated anti-mouse IgG antibody (1:30,000 in dilution buffer) for 1 h at RT. After three additional washes with 300 µL PBST, 100 µL TMB substrate (BioLegend, San Diego, CA, USA) was added to each well and kept in darkness for 15 min. The reactions were stopped with 100 µL 2 N H_2_SO_4_. The optical density at 450 nm was measured with a TECAN spectrophotometer (Mannerdorf, Switzerland).

### 5.4. Hemagglutination Inhibition (HI) and Microneutralization (MN) Assays

For the HI assay, receptor-destroying enzyme (Denka Seiken, Chuo-ku, Tokyo, Japan) treated serum samples were serially diluted two-fold, and incubated with 4 HA units of H7N9 virus (A/Taiwan/01/2013). The treated serum samples were co-incubated with 0.5% turkey red blood cells (RBCs), and HI titers were defined as the reciprocal of the highest dilution completely inhibiting hemagglutination. For the MN assay, two-fold serially diluted serum samples were mixed with equal volumes of H7N9 virus diluent (A/Taiwan/01/2013; 100 TCID50/well) incubated at 4 °C for 1 h, and added to the prepared MDCK cells. Infectivity was defined as the presence of a cytopathic effect observed on day 4. Neutralizing titers were defined as the reciprocals of the highest serum dilutions neutralizing H7N9 virus infectivity in 50% of wells compared to uninfected cells.

### 5.5. Analysis of Antibody-Secreting Cells (ASCs) in the Spleen

ELISPOT assays were used to determine H7HA-specific IgG and IgA ASCs. Splenocytes and CLNs were collected from immunized mice 3 weeks after the final vaccination. Multiscreen 96-well filtration plates (Merck Millipore, Darmstadt, Germany) were coated with rH7 proteins (1 µg per well) and incubated overnight at 4 °C followed by blocking with 200 µL/well of complete RPMI 1640 (10% fetal bovine serum [FBS], 1 × penicillin/streptomycin, 1 × sodium pyruvate, 1 × nonessential amino acids, and 100 µM β-mercaptoethanol) at RT for 1 h. Next, 5 × 10^5^ splenocytes or CLN cells were added to each well and incubated at 37 °C for 48 h. After three washes with PBST, HRP-conjugated anti-mouse IgG or HRP-conjugated anti-mouse IgA was added to each well for IgG- or IgA-ASCs, respectively. After overnight incubation at RT, and PBST and two PBS washes, 3-amino-9-ethylcarbazole substrate (Sigma-Aldrich, St. Louis, MO, USA) was added, followed by incubation at RT for 0.5–1 h before washing with double-distilled water. Immunospots were determined using an ELISPOT plate reader (CTL, Inc., Cleveland, OH, USA).

### 5.6. GC B Cell Analysis Using Flow Cytometry

Flow cytometry analysis (BD Accuri C6, BD Biosciences, San Jose, CA, USA) was used to determine the proliferation of germinal center (GC) B cells as previously described [37]. In brief, 1 × 10^6^ splenocytes per well were stimulated with 20 µg/mL of rH7 proteins for 48 h. Cells were harvested and fixed with 1% formaldehyde for 30 min at 4 °C. Stimulated splenocytes were suspended in staining buffer (2% FBS and 0.01% NaN_3_ dissolved in PBS), simultaneously stained with anti-B220-APC antibodies (BD bioscience, San Jose, CA, USA) and FITC-conjugated peanut agglutinin (PNA) (Sigma-Aldrich, St. Louis, MO, USA, catalog No. L7381), and kept for 30 min at 4 °C. The B220+ PNA+ cells (GC B cells) were gated and analyzed using Accuri C6 software (BD Biosciences).

### 5.7. T Cell Response Analysis 

Splenocytes and CLN cells were seeded at 1 × 10^6^ cells/well and stimulated with 10 μg/mL rH7 proteins for 3 days at 37 °C prior to measuring cytokine levels in culture supernatants collected from stimulated cells. An enzyme-linked immunosorbent assay (ELISA) was used to determine H7HA-specific IFN-γ-, IL-4-, and IL-17A-secreting T cells in splenocytes and CLNs as described in the manufacturer’s instructions. In brief, 96-well plates were coated with anti-mouse IFN-γ, IL-4, or IL17A capture antibodies (eBioscience, San Diego, CA, USA), followed by blocking with PBS buffer plus 1% BSA for 2 h. After washing three times, the coated wells were incubated for 1 h with biotin-conjugated IFN-γ, IL-4, or IL17A detection antibodies, respectively, and another 1 h incubation with avidin-HRP. After this, TMB substrate was added to each well and kept in darkness for 15 min. The reactions were stopped by adding 100 µL 2N H_2_SO_4_. Optical density (450 nm) was measured with a TECAN spectrophotometer.

### 5.8. Virus Challenges

Three weeks after the final immunizations, the mice were anesthetized and intranasally challenged with 50 μL of 10 LD_50_ of the H7N9 virus (A/Taiwan/01/2013). PBS-immunized mice were used as a mock control. Mouse survival and body weights were recorded daily for 14 days. Based on the Institutional Animal Care and Use Committee (IACUC) guidelines, body weight loss >25% was used as an end point. All procedures were approved by the IACUC of Academia Sinica. Per the IACUC guidelines, carbon dioxide was used to sacrifice the mice that survived the challenge experiments to ameliorate suffering. 

### 5.9. Hematoxylin and Eosin (H&E) Staining of Nasal Tissues

Groups of mice (N = 3) were sacrificed one week after the final vaccination. The nasal tissues were harvested, fixed with 10% buffered formalin, and embedded in paraffin. Sections of 5 μm were stained with H&E by the Pathology Core Laboratory of NHRI for histological examination using an Olympus DP70 microscope (Olympus, Shinjuku-ku, Tokyo, Japan) at ×400 magnification.

### 5.10. Immunohistochemical (IHC) Staining for IL-17A Levels in Lung Tissue Cells

Lung tissues were harvested from immunized mice (N = 3) one week after the final immunization. The 5 μm paraffin-embedded sections on silane-coated slides were dewaxed in xylene for 5 min three times, hydrated sequentially in 100%, 95%, 90%, 80%, and 60% ethanol for 5 min each, and then immersed in Trilogy (Cell Marque, Rocklin, CA, USA) at 95°C for 30 min for antigen retrieval. After treating with 3% H_2_O_2_ (in methanol) for 15 min, blocking with 2.5% goat serum (in PBS) for 1 h, and washing with PBS three times, the lung sections were stained with anti-IL-17A (BioLegend) in the dark at RT for 1 h. Finally, the sections were incubated with Simple Stain MAX PO (Histofine, Chuo-ku, Tokyo, Japan) for 1 h, and then treated with DAB Quanto substrate (Thermo Fisher Scientific, Waltham, MA, USA) for 5 min followed by hematoxylin (ScyTck Laboratories, West Logan, UT, USA) counterstaining for 5 min. The sections of each group were observed using an Olympus DP70 microscope at ×400 magnification.

### 5.11. Statistical Analysis

One-way ANOVA with the Holm-Sidak method (GraphPad Prism v6.01, San Diego, CA, USA) was used to analyze the results, with *p* < 0.05 indicating statistical significance. All experiments were performed at least two times each.
